# Fetal development and blood hematological-biochemical parameters in Campeiro and Pantaneiro foals

**DOI:** 10.21451/1984-3143-2017-AR0056

**Published:** 2018-08-16

**Authors:** Gabrielle Sant’anna Vieira, Giane Regina Paludo, Alexandre Floriani Ramos, Ivo Pivato, Rodrigo Arruda de Oliveira

**Affiliations:** 1 Laboratory of Animal Reproduction (*ReproUnB*), , , .; 2 Veterinary Clinical Pathology Laboratory, , , .; 3 Embrapa Recursos Genéticos e Biotecnologia, , .

**Keywords:** equine, fibrinogen, foal, neonatology, ocular

## Abstract

For a long time, Pantaneiro and Campeiro breeds were raised only within their places of origin. Consequently, there are few of these horses; therefore, establishing reproductive and clinical standards for these animals is necessary to implant new biotechnologies for reproduction to preserve their genetics. This study aimed to perform a descriptive evaluation of fetal age determination by fetus ocular orbit measurement in mares of the Campeiro and Pantaneiro breeds. We also evaluated sequential changes in hematology and biochemistry for foals from birth to six months of life by counting red blood cells, hemoglobin concentration, mean corpuscular volume, mean corpuscular hemoglobin concentration, leukocytes, neutrophils, lymphocytes, eosinophils, monocytes, basophils, platelet count, fibrinogen, albumin, plasma protein, creatinine, aspartate aminotransferase, alkaline phosphatase, gamma-glutamyl transferase, and globulins. There was no significant difference in relation to fetal gestational age and ocular orbit between the two breeds (P = 0.578). There was no significant difference in the hematological parameters between the Campeiro and Pantaneiro foals, but there were differences in the means and changes in the blood variables when compared to the literature. These hematological and biochemical variations provide useful information for clinical evaluations from Campeiro and Pantaneiro foals up to six months of age.

## Introduction

Pantaneiro and Campeiro equine breeds are descendants of Iberian horses, which were introduced into Brazil during the colonization period and are known as naturalized breeds. The main characteristics of these breeds are the abilities to acclimatize and multiply under diverse bioclimatic conditions. In addition, these animals play a fundamental role in the herding livestock at the places to which they are inserted, contributing significantly to the economic development of these regions. However, such horses almost disappeared due to an indiscriminate crossing with other breeds, castration of the best animals to work, and diseases ( Santos *et al*., 2007 ).

The first step to starting a conservation of genetic material from Campeiro and Pantaneiro breeds is to assemble data on the reproductive traits of these horses. Therefore, assessing different parameters between the breeds becomes of utmost significance for the establishment of references on horse breeds and peculiarities, which helps reproductive management ( Sereno *et al*., 2008 ; McManus *et al*., 2013 ).

One of the management tools is based on gestational age determination by measuring fetal ocular orbit through ultrasonography ( Turner *et al*., 2006 ). Foal peripartum and hematological profile evaluations before the first feeding until weaning are fundamentally important since each equine species has different hematological values; therefore, studies on these reference intervals and peculiarities of each breed is necessary ( Muñoz *et al*., 2012 ).

Changes in most age-related blood parameters in equine species make the use of reference values impossible for adult animals and in the clinical evaluation of foals from birth to about six months of age ( Medeiros *et al*., 1971 ).

The most accurate knowledge of hematological reference values corresponding to the different breeds and age groups is indispensable for an adequate clinical evaluation of newborn foals ( Harvey *et al*., 1984 ). Nonetheless, using reference values from adult horses to neonatal foals is ineffective for clinical evaluations as long as several hematological parameters change during the first months of life ( Medeiros *et al*., 1971 ).

Therefore, this study aimed to carry out a descriptive evaluation of gestational characteristics, such as fetal age determination by measuring fetus ocular orbit of Campeiro and Pantaneiro mares through ultrasonography. Besides that, it also aimed to evaluate the peripartum of these animals — such as the mare behavior during labor, placental weight, foal height at birth, and blood biochemical profile of foals before the first feeding until weaning.

The findings of this study will set the stage for further studies on other equine breeds from centers for genetic resource conservation, such as Baixadeiro, Lavradeiro, and Marajoara.

## Material and Methods

The experiment was conducted at the Sucupira experimental field station, Embrapa CENARGEN, in Brasilia – Federal District, Brazil. The area lies at latitude 15º46’46”S and longitude 47º 55’46”W. For that, fifteen horses were used (five Campeiro mares and three stallions, and five Pantaneiro mares and two stallions) between 5 and 20 years old (11.4 ± 3.4). The animals were raised in an extensive fodder-feeding system (Mombaça and Tanzania grass), mineral salt, and water *ad libitum* .

### Fetal ocular orbit diameter

After the breeding of ten mares by one of the five stallions, pregnancies were monitored monthly from the third month until the foaling, determining the gestational age through fetal orbit diameter measuring.

These measurements were obtained by rectal ultrasound from where the entire uterus was examined for ocular orbit location. After the orbit was located, an image was frozen to dimension the size of the vitreous body (between the lateral and medial sclera); it consisted of the average between the lateral-medial and rostro-caudal diameters. As for accuracy, three measures were taken for each evaluation ( Turner *et al*., 2006 ).

Once all measurements were completed, the relationship between ocular orbit sizes and gestational age was made to create a formula for regression equation, which could be used in the field for more accurate estimates of the gestational age of equine fetuses, especially for the breeds Campeiro and Pantaneiro.

### Peripartum evaluation

Near the 11th gestation month, mares were separated within a padock of nearly 2,000 m^ 2^ and evaluated daily for attendance at foaling. In this daily control, the mares were restrained in a stock and evaluated from about 50 cm distance. The filling of the udder and teats was assessed and, some hours before delivery, we observed the waxing of teat orifice, which indicated forthcoming delivery ( Silva and Oliveira, 2015 ).

### Placenta weight and foal birth size

At the time of delivery, as soon as neonates stood up in the station, their heights were measured from the front hoof toe to the top of the withers. This measure was obtained using a flexible tape measure. A descriptive evaluation of these values was performed.

Throughout the postpartum period, the mares were monitored through a safe distance of about 10 m for collection and assessment of placenta weight after complete placental delivery. These values were used for a descriptive evaluation of both breeds in this study.

### Blood biochemical parameters

Foal blood sampling was performed shortly after birth, with the first being made immediately after foaling and before colostrum ingestion, and the subsequent ones at 24 h after birth, seven days thereafter, and when the foal was one, two, three, four, five, and six months old. On each occasion, an aliquot of 12 ml blood was collected by jugular venipuncture and divided into one tube with anticoagulant (ethylenediaminetetraacetic acid - EDTA, potassium salt) and another without anticoagulant.

At this stage of the study, we evaluated the sequential changes in hematological parameters (red blood cell counts - RBC, globular volume - GV, hemoglobin - Hb, mean corpuscular volume - MCV, and mean corpuscular hemoglobin concentration - MCHC, leukocyte count - Leuk, leukocyte differential counts - LDC, eosinophils -Eos, monocytes -Mon and platelet counts - PC) and blood biochemistry (urea - U, creatinine - Creat, aspartate aminotransferase - AST, alkaline phosphatase - AP, gamma-glutamyltransferase - GGT, albumin - Alb, total plasma proteins - TPP, fibrinogen - Fib, globulins - Glob, and total serum proteins – Pt). The examinations were carried out at the laboratory of Veterinary Clinical Pathology of the University of Brasília (UnB).

RBC, Leuk, PC, and Hb counts were made using a cell counter (ABX Micros ESV 60 – HORIBA Medical), and the GV and Fib were determined using the microhematocrit method ( Stockham and Scott, 2008 ). The MCV and MCHC were obtained through the standard calculation ( Stockham and Scott, 2008 ). TTP was determined through the refractometer ( Stockham and Scott, 2008 ). From the total, blood smears were made and stained with a rapid dye (panoptic) to perform the LDC and the cellular morphological analysis ( Stockham and Scott, 2008 ). Serum biochemistry (Creat, AST, GGT, AP, and Alb) was determined in a semi-automatic device using specific biochemical kits (BioPlus2000 – BioPlus, Brazil^2^ Labtest®, Brazil).

Glob values were obtained by subtracting the serum proteins from the Alb (Pt-Alb = Glob) ( Stockham and Scott, 2008 ).

### Statistical analysis

In order to obtain the statistical analysis of the relationship between fetal ocular orbital and gestational age, a polynomial regression model was run by R statistical software (RX64 3.3.0), and the comparison between the regression models of both breeds was performed by a covariance analysis test (ANCOVA). This procedure fits up to ten different regression models for two variables: one dependent and one independent.

For the sequential changes in hematological parameters over time, descriptive statistics methods were used, including dispersion, standard deviation, and variance (One-way ANOVA test). The growth curves for each blood parameter, between breeds, were compared through a curve growth analysis, also using R statistical software (RX64 3.3.0). After verifying no significant difference in the results regarding blood parameters between Campeiro and Pantaneiro foals, the dataset of both breeds was combined and comparisons for each sampling time were made through the Welch’s two-sample t-test with the same software R (RX64 3.3.0).

## Results

**Figure 1:**
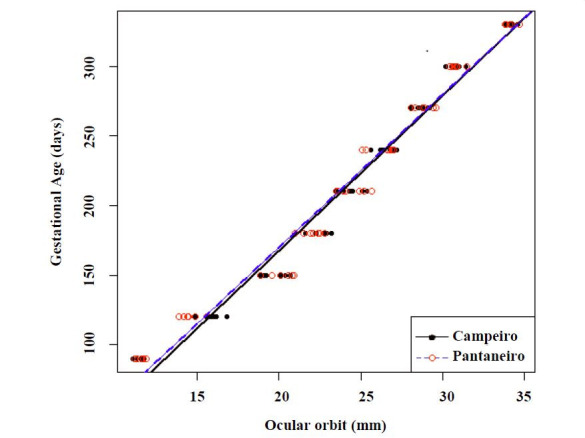
Relationship between fetal ocular orbit diameter and gestational age in 14 mares of Campeiro (n = 7) and Pantaneiro (n = 7) breed, from December 2014 to March 2016.

The best model to relate fetal ocular orbit measurements and gestation age was a linear regression. For Campeiro, the model was *y* = -55.802 + 11.1*x* (R^ 2^ = 0.977; P < 0.05) and for Pantaneiro was *y* = -49.742 + 10.9* x* (R^2^ = 0.972; P < 0.05), wherein *y* represents the gestational age in days and *x* represents the fetal ocular orbit diameter in millimeters, as shown in Figure 1 .

When comparing Campeiro and Pantaneiro breed regression models by ANCOVA test, the relationship between gestation age and fetal ocular orbit diameter showed no significant differences (P = 0.578). Therefore, both models could be used for either Pantaneiro or Campeiro breeds to estimate gestational age. Table 1 shows the placenta weights and animal heights for Campeiro and Pantaneiro foals. Table 2 displays the values of GV, MCV, MCHC, RBC, Hb, Leuk, Lymph, Eos, Mon, TTP, Fib, and PC of Campeiro and Pantaneiro foals from birth (D0) before the first feeding until weaning at 180 days of life. Table 3 describes the biochemical parameters related Alb and Glob concentrations; GGT, AP, and AST activities; Creat and U serum concentration for Campeiro and Pantaneiro foals from birth (day 0) before the first feeding until weaning at 180 days of life.

**Table 1:** Minimum and maximum values (Mean ± SD) of placenta weight (kg) and neonate heights for Campeiro (n6) and Pantaneiro (n = 6) foals.

Breed	Parameter	Min – Max	Mean ± SD
Campeiro	Placenta Weight (kg)	3.4 – 4	3.69 ± 0.21
	Foal birth height (cm)	58 – 72	62 ± 5.54
Pantaneiro	Placenta Weight (kg)	3.3 – 4.2	3.88 ± 0.31
	Foal birth height (cm)	59 – 70	62.2 ± 4.53

**Table 2:** Mean hematological values ± Standard deviation of Campeiro (n = 6) and Pantaneiro (n = 6) foals, from the birth (D 0) before the first feeding until weaning (180 days of life).

	Day 0	Day 1	Day 7	Day 30	Day 60	Day 90	Day 120	Day 150	Day 180
GV (%)	49.58 ± 1.4^A^	48.5 ± 0.86^B^	39.1 ± 0.97^B^	40.79 ± 1.3^B^	37.95 ± 1.08^B^	36.37 ± 1.73^B^	36.93 ± 1.72^B^	35.99 ± 1.03^B^	36.32 ± 0.73^B^
MCV (fL)	45.18 ± 1.92^A^	43.33 ± 1.6^B^	41.17 ± 1.64^B^	39.12 ± 1.51^B^	36.29 ± 1.51^B^	36.64 ± 2.08^B^	35.82 ± 0.86^B^	36.1 ± 0.5^B^	37.47 ± 0.62^B^
MCHC (g/dl)	37.2 ± 1.8^A^	34.1 ± 1.6^B^	34.6 ± 1.5^B^	33.1 ± 1.6^B^	32.8 ± 0.7^B^	34.3 ± 1.0^B^	34.3 ± 1^B^	35.5 ± 1.3^B^	35.3 ± 2.1^B^
RBC (x10^2^/L)	11.26 ± 0.76^A^	10.56 ± 1.06^A^	9.8 ± 0.98^B^	9.39 ± 0.98^B^	9.5 ± 0.85^B^	8.11 ± 0.83^B^	9.66 ± 1.08^B^	10.15 ± 0.86 ^B^	8.97 ± 0.9^B^
Hb (g/dl)	15.1 ± 0.9^A^	15.5 ± 0.8^A^	13.9 ± 1.1^B^	12.1 ± 1.1^B^	12.1 ± 0.6^B^	11.8 ± 1.0^B^	12.6 ± 1.08^B^	12.8 ± 0.8^B^	12.1 ± 1.5^B^
Leuk (x10^9^/L)	5.93 ± 0.52^A^	6.9 ± 0.56^B^	7.23 ± 0.38^B^	7.71 ± 0.66^B^	8.91 ± 0.96^B^	10.4 ± 1.26^B^	11.72 ± 0.81^B^	12.1 ± 0.64^B^	11.29 ± 0.61^B^
Lymph (x10^6^/L)	1488.5 ± 135.1^A^	2122.5 ± 316.1^B^	2255.8 ± 473.4^B^	2786.3 ± 310.5^B^	3088.8 ± 346.7^B^	2803.7 ± 201.5^B^	3366.7 ± 689.0^B^	4133.7 ± 798.0^B^	4466.9 ± 896.0^B^
Neu (x10^6^/L)	4108 ± 549^A^	4307 ± 406^A^	4450 ± 719^A^	4365 ± 696^A^	5170 ± 769^B^	6784 ± 1261^B^	7640 ± 851^B^	7046 ± 1007^B^	5819 ± 1076^B^
Mon (x10^6^/L)	212.3 ± 109.6^A^	273.5 ± 73.5^A^	368.9 ± 61.2^B^	352.1 ± 81.9^B^	495.5 ± 139^B^	641.1 ± 153.7^B^	497.1 ± 193.5^B^	779.1 ± 251.1^B^	778.3 ± 103.8^B^
Eos (x10^6^/L)	124.1 ± 27.6^A^	140.6 ± 40.7^A^	162.3± 36.4^B^	205 ± 49.3^B^	161.9 ± 50.7^B^	175.6 ± 56.9^B^	220.6 ± 52.8^B^	200.4 ± 94.9^B^	226.2 ± 61.3^B^
PC (x10^3^/l)	242741 ± 29960^A^	207372 ± 46079^B^	223477 ± 35690^B^	297086 ± 90908^B^	289103 ± 53303^B^	260713 ± 40793^B^	275500 ± 42539^B^	261430 ± 49808^B^	309175 ± 41095^B^
TPP (g/dl)	4.59 ± 0.3^A^	7.14 ± 1.01^B^	7.3 ± 0.69^B^	7.45 ± 0.3^B^	6.62 ± 0.25^B^	6.6 ± 0.36^B^	7.05 ± 0.42^B^	6.9 ± 0.39 ^B^	6.82 ± 0.51^B^
Fib (mg/dl)	233.3 ± 58.9^A^	341.6 ± 60.6^B^	391.6 ± 49.3^A^	354.1 ± 74.8^B^	341.6 ± 53.3^B^	362.5 ± 46.2^B^	337.5 ± 54.4^B^	320.8 ± 51.8^B^	287.5 ± 61.6^B^

A Different letters represent significant differences (P < 0.05) regarding globular volume (GV), mean corpuscular volume (MCV), mean corpuscular hemoglobin concentration (MCHC), red blood cells (RBC), hemoglobin concentration (Hb), leukocytes (Leuk), lymphocytes (Lymph), neutrophils (Neu), monocytes (Mon), eosinophils (Eos), platelets count (PC), total plasma proteins (TPP), and fibrinogen (Fib).

**Table 3:** Biochemical mean values ± standard deviation for Campeiro (n=6) and Pantaneiro (n=6) foals from birth (D0) before the first feeding until weaning at180 days of life.

	Day 0	Day 1	Day 7	Day 30	Day 60	Day 90	Day 120	Day 150	Day 180
Alb (g/dl)	2.07 ± 0.16^A^	2.9 ± 0.14^B^	3.2 ± 0.16^B^	3.9 ± 0.29^B^	2.6 ± 0.22^B^	2.9 ± 0.18^B^	3.2 ± 0.28^B^	3.2 ± 0.22^B^	2.5 ± 0.31^A^
Glob (g/dl)	4.5 ± 0.4^A^	4.2 ± 1.0^A^	4.4 ± 0.7^A^	3.9 ± 0.7^B^	3.6 ± 0.7^B^	3.7 ± 0.6^B^	4.2 ± 0.8^A^	3.7 ± 0.6^B^	3.7 ± 0.4^B^
Pt (g/dl)	6.6 ± 0.5^A^	7.1 ± 1.1^B^	7.6 ± 0.9^B^	7.81 ± 0.8^B^	6.2 ± 1.1 ^A^	6.6 ± 0.7^A^	7.4 ± 1.2^B^	6.9 ± 0.5^B^	6.2 ± 0.8^A^
GGT (U/L)	19.42 ± 2.28^A^	35.83 ± 4.15^B^	26.16 ± 1.77^B^	20.75 ± 5.53^B^	20.58 ± 5.43^B^	18.08 ± 2.95^B^	14.7 ± 1.32^B^	21.5 ± 1.04^B^	20.66 ± 2.39^A^
AP (U/L)	1661 ± 164^A^	1691 ± 399^A^	903 ± 231^B^	441 ± 60^B^	493 ± 67^B^	540 ± 62^B^	479 ± 122^B^	471 ± 23^B^	585 ± 360^B^
AST (U/ml)	94.6 ± 25.2^A^	189.8 ± 33.2^B^	233.8 ± 25.4^B^	247.1 ± 61.7^B^	219 ± 52.6^B^	213.8 ± 32.9^B^	250 ± 48.6^B^	251.2 ± 59.8^B^	259.4 ± 30.3^B^
Creat (g/dl)	2.6 ± 0.23^A^	1.67 ± 0.43^B^	1.22 ± 0.36^B^	1.15 ± 0.21^B^	1.28 ± 0.21^B^	1.18 ± 0.24^B^	1.11 ± 0.17^B^	1.18 ± 0.12^B^	1.0 ± 0.16^B^
U (mg/dl)	17.91 ± 1.11^A^	23.83 ± 2.3^B^	18.66 ± 5.49^A^	19.25 ± 5.8 ^B^	20.91 ± 4.31^B^	22.41 ± 2.28^B^	21.83 ± 1.77^B^	24.66 ± 3.92^B^	20.16 ± 1.72^B^

A Different letters represent significant differences (P < 0.05) when referring to albumin (Alb), globulin (Glob), total serum proteins (Pt), gamma-glutamyltransferase (GGT), alkaline phosphatase (AP), aspartate aminotransferase (AST), creatinine (Creat), and urea (U).

## Discussion

Our study revealed a direct relationship between fetal ocular orbit diameter and gestation age, which is a reliable parameter to estimate the gestation time in equine species. Moreover, the sameness of results between the two studied breeds can be explained by similarities in origin and size of these animals. Both breeds descend from Iberian breeds introduced into the central-south of Brazil, brought by Spanish expeditions during the 18th century ( Santos *et al*., 1992 ).

A positive relationship was found between placental weight and foal birth height, corroborating the findings of some authors ( Allen *et al*., 2002 ; Elliott *et al*., 2009 ; Fernandes *et al*., 2012 ; Meireles *et al.*, 2017). According to Wilsher and Allen (2003) , the birth of heavier and larger foals can be explained by the fact that heavy placentas are more likely to cause uterine distention in mares. Therefore, as equine placentas are classified as diffuse epithelial, it becomes connected to the entire uterine surface, and the larger its mass, the greater the absorption of nutrients and gases; consequently, the greater the size and weight of the foal at birth. Both the mean values of Campeiro and Pantaneiro foal heights and the average values of placenta weights were very close, once again because these animals had similar origins and characteristics.

Regarding the blood variables, for both breeds, GV, MCV, and Hb presented values above the average established by Harvey *et al*. (1984) . These authors created a reference for foals within the studied age range. These high values of GV, MCV, and Hb observed in our study might have been due to dehydration since the studied horses were in a region with a period of intense drought and in extremely large pastures, what makes water intake difficult for younger animals. These high values can also be attributed to possible physiological adaptations of these animals to the region of hot and dry climate, which are distinct from the weather where these breeds originated and are still present today.

Except for MCHC, the erythrogram results for Campeiro and Pantaneiro foals differed from the findings of Ribeiro *et al*. (2008) and Fonteque *et al*. (2016) , who studied juvenile and adult equines of the same breeds. These authors reported low erythrocyte measurements if compared to other breeds. The explanation for this difference between young animals of the same breed may be the differences in climate, temperature, food availability, and animal excitability during the experiment period. The animals studied by Ribeiro *et al*. (2008) were located in the Pantanal region of the State of Mato Grosso (Brazil), where horses spend much of the year in wetlands with high humidity, abundant food, and water. On the other side, the foals studied here were in the Federal District, a region with a very dry climate, high temperatures, and low food availability for much of the year.

According to Grondin and Dewitt (2010) , pure blood horses such as the Andalusian and Lusitano present high erythrocyte, Hb, and average GV values when compared to animals of other breeds. Therefore, again, one possible explanation for the high values of GV, MCV, and Hb in Campeiro and Pantaneiro breeds can be attributed to their Iberian origin — brought during the Brazilian colonization period.

From birth to 180 days of life, the MCHCs were below the references by Harvey *et al*. (1984) . This outcome might be related to a likely iron deficiency since mares grazed pastures poor in minerals during gestation, in addition to a low iron availability within the neonate first days. According to that author, newborn animals and children are prone to develop iron deficiency when compared to adults, as there is a limitation of body iron stores, as well as an increase in iron demand for fast growth of neonates, and a decrease in this element concentration in breast milk.

Campeiro and Pantaneiro foals up to 8 months old presented Leuk and Neu counts above the reference obtained from adult animals over 25 months, being significantly above the results described by other authors who studied foals of the same age ( Ribeiro *et al*., 2008 ; Fonteque *et al*., 2016 ). Among the related causes, the most consistent is due to the stress experienced by these animals throughout the experiment, from postpartum to 6 months of age. In this sense, the increase in the number of leukocytes after exercise, generally ranging from 10 to 30%, is related to an elevation in cortisol levels ( Snow *et al*., 1982 ).

Lymph counts in locally adapted Brazilian foals, between 30 and 180 days of age, fell below to those reported by Harvey *et al*. (1984) . Lymph data for Brazilian animals are in concordance with those of Jeffcott *et al*. (1982) , who associated an increase in Lymph cell counts within the first months of life to a constant development of the lymphatic system after foal birth.

During the 6 months of the experiment, plasma globulin averages remained higher than the reference values described by Bauer *et al*. (1990), except for the moment before colostrum ingestion since there are no references in the literature about Glob during this period. Hyperglobulinemia may be observed in uterine antigen stimulation resulting from a possible infection or inflammation (Bauer *et al*., 1990), but in spite of this finding, no clinical alteration was observed in these animals.

Both breeds presented U measurements higher than the mean established as a reference for foals between 24 h and 180 days described by Harvey *et al*. (1984) . On the other hand, Creat was within the references for foals and adult animals throughout the study. Bauer *et al*. (1984) assigned the high value of U measurements to a negative energy balance — when body tissues are metabolized to generate energy, especially in young animals — a relatively common finding in foals under some type of stress and/or intrauterine catabolism. This explanation can be attributed to the high U measurements observed in Campeiro and Pantaneiro foals, especially after delivery, since most of the mares went through stress conditions in the days prior to delivery and particularly at the moment of birthing, probably due to the daily evaluation to predict and monitor the delivery.

Between 24 h postpartum and 180 days, AP averages remained higher than were those described by Bauer *et al*. (1984) , which is a reference for horses in this age group. These high values of AP during the early days of life can be attributed to an intense osteoblastic activity related to bone growth and intestinal phagocytic functioning during the first 24 h of equine neonates ( Rumbaugh and Adamson, 1983 ).

Fetal ocular-orbit growth in Campeiro and Pantaneiro mares followed a linear pattern, developing two regression models with no differences from one another.

Campeiro and Pantaneiro foals were similar regarding hematological and biochemical parameters from birth (before colostrum intake) until 180 days of life. Yet, both breeds presented different results when compared to the literature. As a result, these characteristics should be taken into account when interpreting the laboratory results for blood variables.

## Ethical Approval

The Committee of Ethics in Animal Research of EMBRAPA, Brasilia, Federal District (protocol n. 009-2015), approved this study.
